# Efficacy of psychological interventions versus standard care for cancer pain: a systematic review and meta-analysis

**DOI:** 10.3389/fpsyt.2026.1811605

**Published:** 2026-09-16

**Authors:** Yue Hu, Hai-Xiao Liu, Ling-Yun Wang, Chang-Lin Li, Dong-Tao Li, Ling-Han Meng, Guang-Da Zheng, Yan-Ju Bao

**Affiliations:** 1 Department of Second Clinic, Guang’anmen Hospital, China Academy of Chinese Medical Sciences, Beijing, China; 2 Department of Pharmacy, The First Clinical College, Liaoning University of Traditional Chinese Medicine, Shenyang, China

**Keywords:** beck depression inventory, cancer-related pain, psychological intervention, randomized controlled trial, visual analog scale

## Abstract

**Background:**

Cancer-related pain is frequently accompanied by psychological distress, which may worsen symptom burden and impair quality of life. This systematic review and meta-analysis aimed to evaluate the efficacy of psychological interventions versus standard care for pain intensity, depressive symptoms, and anxiety in adults with cancer-related pain.

**Methods:**

This systematic review and meta-analysis followed the Preferred Reporting Items for Systematic Reviews and Meta-Analyses (PRISMA) guidance. PubMed, Embase, Web of Science, and the Cochrane Library were searched from inception to January 5, 2026. Randomized controlled trials comparing structured psychological interventions with routine or standard care were included. The primary outcome was pain intensity measured by the Visual Analog Scale (VAS). Secondary outcomes were depressive symptoms measured by the Beck Depression Inventory (BDI) and anxiety measured by the State-Trait Anxiety Inventory (STAI). Mean differences (MDs) with 95% confidence intervals (CIs) were pooled.

**Results:**

Fourteen randomized controlled trials involving 2, 070 participants were included. Psychological interventions significantly reduced VAS scores compared with standard care (MD = −0.91, 95% CI −1.38 to −0.44; P = 0.0001; I²=97%). Significant improvements were also observed in BDI scores across 10 trials (MD = −4.22, 95% CI −5.50 to −2.93; P<0.00001; I²=78%) and STAI scores across 5 trials (MD = −1.74, 95% CI −2.96 to −0.52; P = 0.005; I²=0%). Subgroup analyses by cancer type and mean age partly explained heterogeneity in VAS outcomes. Egger’s tests showed no significant funnel plot asymmetry for VAS (P = 0.857) or BDI (P = 0.452). Adverse events and follow-up durations were inconsistently reported and could not be quantitatively synthesized. The certainty of evidence was moderate for all outcomes.

**Conclusion:**

Psychological interventions may reduce pain intensity, depressive symptoms, and anxiety in adults with cancer-related pain. However, substantial heterogeneity, inconsistent follow-up reporting, and limited safety data warrant cautious interpretation and further standardized randomized trials.

## 1 Introduction

Cancer pain is one of the most debilitating symptoms in patients with malignancies, affecting approximately 50%–70% of patients during active treatment and nearly one-third of cancer survivors (1–3). It is often chronic and multifactorial, resulting from tumor progression, treatment-related toxicity, inflammatory processes, and nociceptive or neuropathic mechanisms (4, 5). Persistent pain can impair physical function, aggravate emotional distress, disrupt social relationships, reduce adherence to oncologic treatment, and ultimately compromise quality of life (6, 7). Psychological factors are closely involved in the development and persistence of cancer-related pain (8). Anxiety and depression may amplify pain perception through dysregulation of the hypothalamic-pituitary-adrenal axis and descending pain-modulatory pathways, while uncontrolled pain can further intensify psychological distress (9, 10). Therefore, psychological interventions have been proposed as important adjuncts to pharmacological analgesia. By modifying maladaptive cognitions, improving emotional regulation, and strengthening coping skills, interventions such as cognitive-behavioral therapy, acceptance and commitment therapy, mindfulness-based approaches, and psychoeducational support may help interrupt the pain-distress cycle and improve patient-centered outcomes (11, 12). However, their effectiveness across different cancer populations remains incompletely characterized and requires systematic evaluation (13–15).

A comprehensive assessment of psychological interventions should include both pain intensity and related psychological symptoms. Pain intensity reflects the core symptom burden, whereas depression and anxiety are clinically important because they are highly prevalent among patients with cancer-related pain and may worsen pain perception, treatment response, and overall prognosis (16–18). Standardized instruments allow consistent outcome assessment across studies. The Visual Analog Scale (VAS) is widely used to measure pain intensity, while the Beck Depression Inventory (BDI) and State-Trait Anxiety Inventory (STAI) are validated tools for assessing depressive and anxiety symptoms, respectively (19, 20). Cancer-related pain is increasingly recognized as a multidimensional symptom influenced not only by nociceptive and disease-related mechanisms but also by emotional, cognitive, and behavioral factors (21). Previous studies and reviews have suggested that psychological and behavioral interventions may contribute to cancer pain management, and some evidence has also supported their role in alleviating psychological distress among patients with cancer (22, 23). However, existing syntheses have often focused on pain outcomes alone, specific cancer populations, advanced disease settings, or broader non-pharmacological interventions, leaving uncertainty regarding the overall effect of structured psychological interventions on both pain intensity and coexisting depressive and anxiety symptoms in adults with cancer-related pain (24, 25).

Therefore, an updated synthesis restricted to randomized controlled trials and integrating patient-reported pain, depression, and anxiety outcomes, together with assessment of heterogeneity, publication bias, and certainty of evidence, may provide a more clinically relevant summary of the current evidence. To address this gap, we conducted a systematic review and meta-analysis to evaluate the efficacy of psychological interventions compared with standard care for reducing pain intensity, depressive symptoms, and anxiety in adult patients with cancer-related pain.

## 2 Materials and methods

### 2.1 Data sources and retrieval

This meta-analysis followed the Preferred Reporting Items for Systematic Reviews and Meta-Analyses (26). The PubMed strategy was developed using a combination of Medical Subject Headings (MeSH) and free-text terms and then adapted for other databases using their respective controlled vocabularies and search interfaces (Emtree for Embase and topic field searching for Web of Science). Boolean operators, truncation, and proximity operators were applied as appropriate for each database. No filters for language or publication year were applied during the initial search. The detailed search strategies for all databases are provided in **Supplementary Table 1**.

### 2.2 Eligibility criteria

Studies were included according to the PICOS framework. Eligible studies met the following criteria:

1. Population: adult patients aged 18 years or older with cancer-related pain caused by malignancy or cancer treatment;
2. Intervention: structured psychological interventions, including cognitive-behavioral therapy, mindfulness-based interventions, acceptance and commitment therapy, relaxation-based therapy, coping skills training, supportive psychotherapy, or psychoeducational interventions. The intervention had to include a clear psychological, behavioral, emotional regulation, or coping-related component;
3. Comparator: routine or standard care without structured psychological intervention. Standard care was defined as usual oncology care, routine pain management, analgesic treatment, nursing care, health education, or regular follow-up provided according to local clinical practice;
4. Outcomes: at least one relevant outcome, including pain intensity measured by the VAS (27), depressive symptoms measured by the BDI (28), or anxiety measured by the STAI (29);
5. Study design: randomized controlled trials.

Studies were excluded if they were reviews, editorials, case reports, conference abstracts without sufficient data, non-randomized studies, or studies involving pediatric populations. Studies focusing on pain conditions not primarily related to cancer were also excluded. Trials in which psychological interventions were combined with other non-psychological therapies and the independent effect of the psychological component could not be isolated were excluded. Studies without available full text or without sufficient quantitative data for meta-analysis were excluded after retrieval efforts. When multiple publications reported overlapping populations, the report with the most complete dataset or the longest follow-up was selected.

### 2.3 Study selection and data extraction

Two reviewers independently screened all retrieved records. After duplicate removal, titles and abstracts were reviewed first to exclude clearly irrelevant studies. Potentially eligible articles were then assessed by full-text review according to the predefined inclusion and exclusion criteria. Reasons for exclusion at the full-text stage were recorded. Any disagreement during study selection was resolved through discussion, and unresolved discrepancies were adjudicated by a third reviewer. Data were extracted independently by two reviewers using a standardized data extraction form. The extracted information included first author, publication year, country or region, study design, cancer type, sample size, patient age and sex, intervention type, intervention duration and delivery format, control condition, follow-up duration, outcome measures, and numerical data required for meta-analysis. For continuous outcomes, means, standard deviations, and sample sizes were extracted when available. Disagreements in data extraction were resolved by consensus or consultation with a third reviewer.

### 2.4 Risk-of-bias assessment

The methodological quality of the included RCTs was assessed independently by two reviewers using the Cochrane Risk of Bias tool (RoB 1.0). The following domains were evaluated: random sequence generation, allocation concealment, blinding of participants and personnel, blinding of outcome assessment, incomplete outcome data, selective outcome reporting, and other potential sources of bias. Each domain was judged as “low risk, ” “high risk, ” or “unclear risk” according to the criteria recommended in the Cochrane Handbook. Disagreements were resolved by discussion or consultation with a third reviewer.

### 2.5 Statistical analysis

Meta-analysis was performed using Review Manager 5.3. Continuous outcomes were summarized as mean differences (MDs) with 95% confidence intervals (CIs). Pain intensity measured by VAS was defined as the primary outcome, while depressive symptoms measured by BDI and anxiety measured by STAI were secondary outcomes. Statistical heterogeneity was assessed using Cochran’s Q test and the I² statistic. A fixed-effect model was used when heterogeneity was not substantial (P≥0.10 and I²≤50%); otherwise, a random-effects model was applied. When substantial heterogeneity was detected, subgroup analyses were conducted to explore potential sources of between-study variability, including cancer type and mean patient age. Publication bias was assessed for outcomes including at least 10 studies using funnel plots. Egger’s regression test was further performed using Stata 17.0 to quantitatively evaluate funnel plot asymmetry, with P<0.10 indicating potential small-study effects. For pooled effect estimates, a two-sided P value <0.05 was considered statistically significant.

### 2.6 Certainty of evidence assessment

The certainty of evidence for each outcome was assessed using the Grading of Recommendations Assessment, Development and Evaluation approach. Evidence from randomized controlled trials was initially rated as high certainty and was downgraded when important limitations were identified across the prespecified GRADE domains. The final certainty of evidence was classified as high, moderate, low, or very low. The GRADE assessment was performed independently by two reviewers, and disagreements were resolved through discussion or consultation with a third reviewer.

## 3 Results

### 3.1 Literature search and study selection

A total of 11, 446 records were initially identified through database searches, including 1, 599 from PubMed, 1, 886 from Web of Science, 2, 381 from the Cochrane Library, and 5, 580 from Embase. After removing 954 duplicate records, 10, 492 records remained for title and abstract screening. During the initial screening stage, 10, 161 records were excluded for the following reasons: mismatch in research design (n=2, 921), mismatch in research population (n=3, 853), absence of relevant outcome indicators (n=3, 332), serious risk of bias (n=44), and non-English publication (n=11). A total of 331 reports were sought for retrieval, of which 4 could not be obtained. Therefore, 327 full-text reports were assessed for eligibility. Among them, 313 reports were excluded because of unavailable or unsuitable data (n=201) or inappropriate study design (n=112). No additional records were identified through websites, organizations, citation searching, or other sources. Ultimately, 14 studies were included in the systematic review and meta-analysis (30–43). The detailed literature selection process is presented in **Figure 1**.

**Figure 1:**
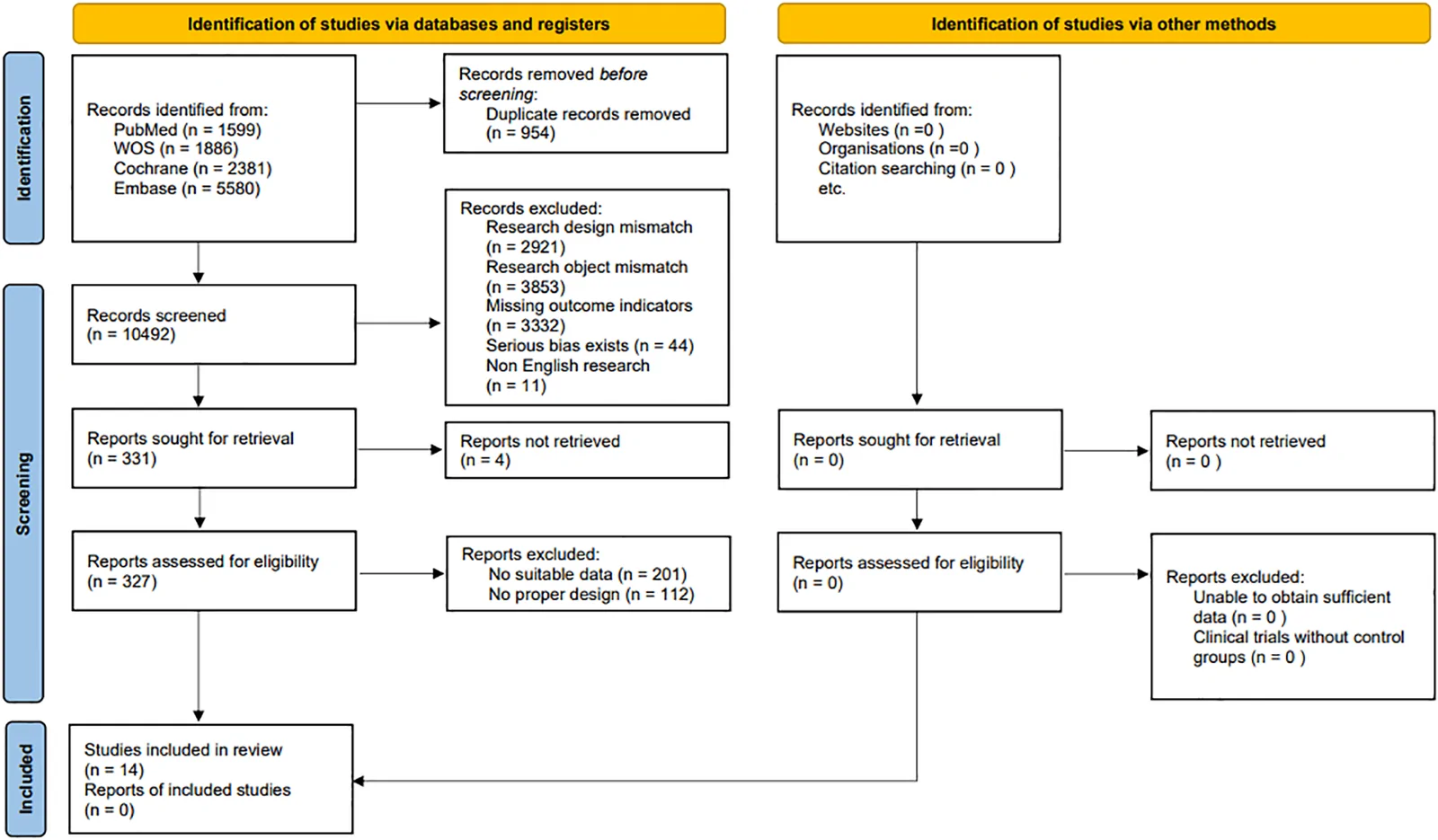
PRISMA flow diagram of literature search and study selection.

### 3.2 Characteristics of the included studies

A total of 14 RCTs (30–43) were included in this meta-analysis, all of which evaluated the effects of psychological interventions on cancer-related pain in adult patients. The included studies involved various cancer types, including breast cancer in 6 studies, gastrointestinal cancer in 2 studies, colon cancer in 2 studies, prostate cancer in 1 study, osteosarcoma in 1 study, and unspecified cancer types in 2 studies. The sample size ranged from 40 to 376 participants per study, and the median age across the treatment and control groups ranged from 47 to 61 years. The psychological interventions varied across studies and mainly included cognitive-behavioral therapy, mindfulness-based interventions, and psychoeducational support. The control groups received routine or standard care without structured psychological intervention. The main outcomes were pain intensity assessed using VAS, depressive symptoms assessed using BDI, and anxiety assessed using STAI. The basic characteristics of the included studies are summarized in **Table 1**.

**Table 1:** Basic characteristics of the included studies (n = 14).

Include studies	Country	Research subject	Cancer type	Number		Age		Intervention	Control	Outcome measure
				T	C	T	C			
Arefian, M. 2025 (31)	Iran	cancer pain	breast cancer	21	21	45	47	psychological intervention	routine intervention	①②
Arefian, M. 2024 (30)	Iran	cancer pain	breast cancer	21	21	45	47	psychological intervention	routine intervention	①②
Dams, L. 2023 (32)	Belgium	cancer pain	breast cancer	92	92	55	55	psychological intervention	routine intervention	①②
Duzova, U. S. 2025 (33)	Turkey	cancer pain	breast cancer	40	40	59	58	psychological intervention	routine intervention	①③
Giannelli, A. 2024 (34)	Italy	cancer pain	gastrointestinal cancer	27	26	55	53	psychological intervention	routine intervention	①②③
Harper, F. W. K. 2023 (35)	USA	cancer pain	/	376	332	60	60	psychological intervention	routine intervention	①②
Kaplan, M. 2025 (36)	Turkey	cancer pain	colon cancer	35	35	59	57	psychological intervention	routine intervention	①②
Namazinia, M. 2023 (37)	Iran	cancer pain	gastrointestinal cancer	34	35	49	45	psychological intervention	routine intervention	①③
Oz Kahveci, S. 2025 (38)	Turkey	cancer pain	Osteosarcoma	20	19	48	44	psychological intervention	routine intervention	①③
Rajabi, F. 2025 (39)	Iran	cancer pain	/	60	60	46	48	psychological intervention	routine intervention	①②
Ren, Y. 2025 (40)	China	cancer pain	breast cancer	144	143	48	47	psychological intervention	routine intervention	①②
Wang, G. 2025 (41)	China	cancer pain	colon cancer	109	109	52	53	psychological intervention	routine intervention	①③
Winger, J. G. 2023 (42)	USA	cancer pain	prostate cancer	30	30	61	60	psychological intervention	routine intervention	①②
Zhang, F. 2025 (43)	China	cancer pain	breast cancer	47	51	52	53	psychological intervention	routine intervention	①②

T, treatment group; C, control group; ① Visual Analog Scale; ② Beck depression Rating Scale; ③ State-Trait Anxiety Inventory.

### 3.3 Risk of bias assessment

The risk of bias of the included RCTs was assessed using the Cochrane Risk of Bias tool. The results are summarized in **Figure 2**. Overall, the most frequent methodological concerns were related to blinding of participants and personnel and blinding of outcome assessment. These concerns were expected because psychological interventions are difficult to blind in clinical practice. However, because the main outcomes, including VAS, BDI, and STAI scores, were self-reported, inadequate blinding may have increased the risk of performance and detection bias. Some studies also provided insufficient details regarding allocation concealment, resulting in an unclear risk of selection bias. In contrast, most studies reported acceptable completeness of outcome data, and the risk related to incomplete outcome data was generally low. Selective reporting was judged as low or unclear risk in most studies, depending on whether prespecified outcomes were sufficiently reported. No study was excluded based on risk of bias alone. Nevertheless, unclear or high-risk domains, particularly those related to blinding, may have influenced the magnitude of the pooled effects and contributed to between-study heterogeneity.

**Figure 2:**
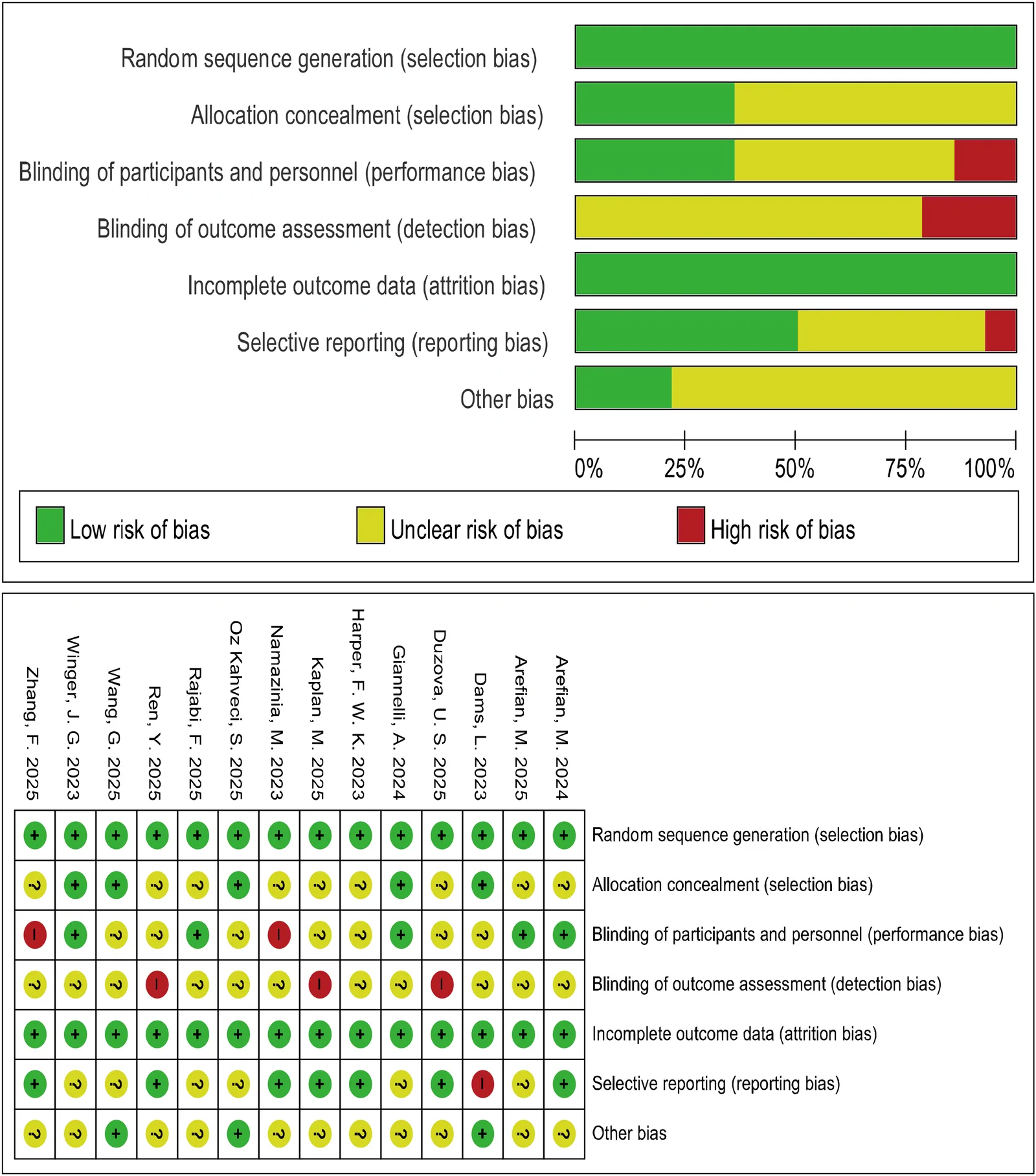
Results of bias risk assessment of the included studies.

### 3.4 Meta analysis results

#### 3.4.1 VAS

A total of 14 studies (30–43) were included in the analysis of VAS scores. Significant heterogeneity was observed among the included studies (P<0.00001, I²=97%); therefore, a random-effects model was applied. The pooled results showed that psychological interventions were associated with significantly lower VAS scores compared with routine care (MD = −0.91, 95% CI −1.38 to −0.44; P = 0.0001; **Figure 3**).

**Figure 3:**
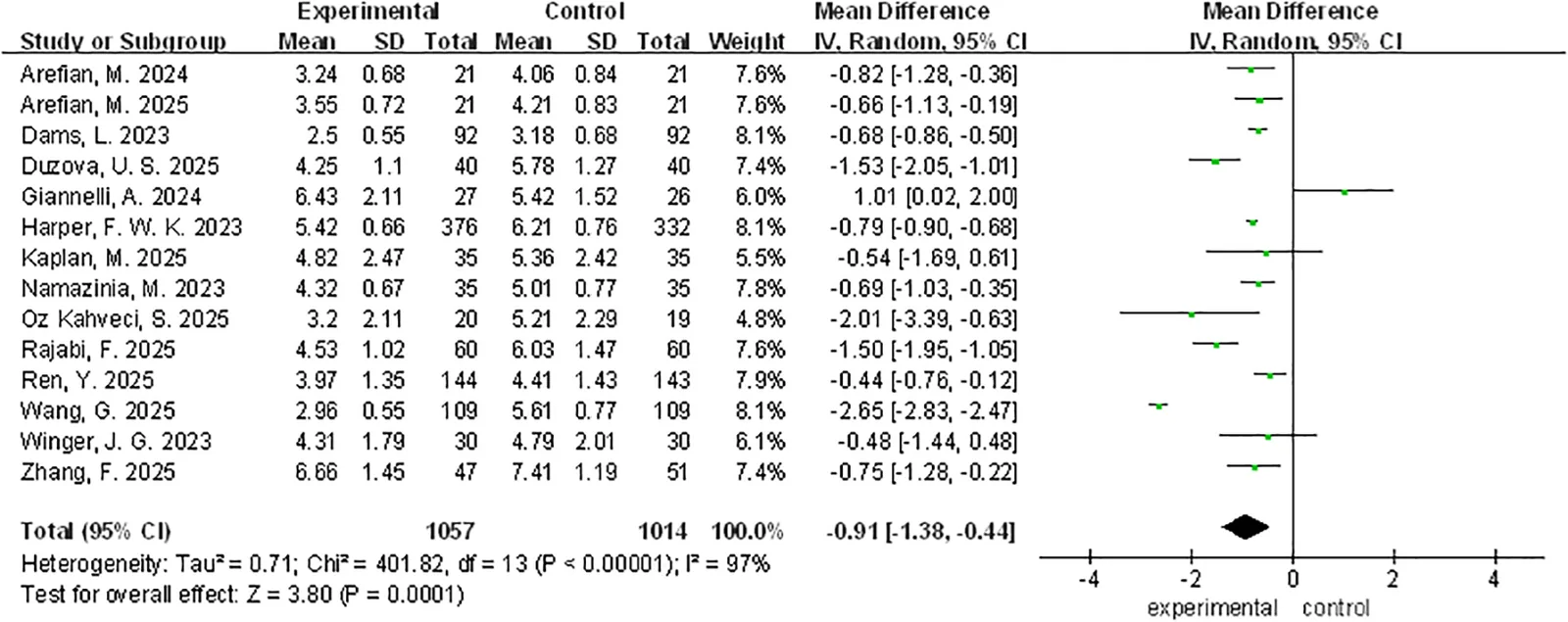
Meta-analysis of VAS comparison between psychological intervention group and routine intervention group.

#### 3.4.2 BDI

A total of 10 studies (30–32, 34–36, 39, 40, 42, 43) were included in the analysis of BDI scores. Substantial heterogeneity was observed among the studies (P<0.00001, I²=78%); therefore, a random-effects model was used. The pooled results showed that psychological interventions were associated with significantly lower BDI scores compared with routine care (MD = −4.22, 95% CI −5.50 to −2.93; P<0.00001; **Figure 4**).

**Figure 4:**
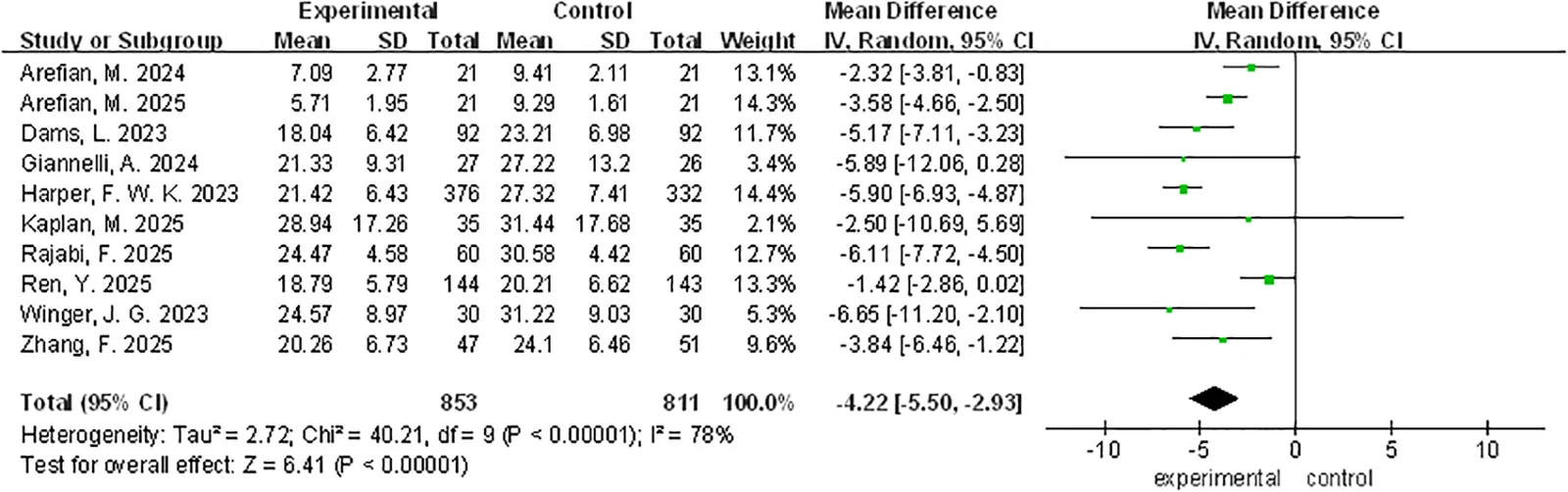
Meta-analysis of BDI comparison between psychological intervention group and routine intervention group.

#### 3.4.3 STAI

A total of 5 studies (33, 34, 37, 38, 41) were included in the analysis of STAI scores. No significant heterogeneity was observed among the studies (P = 0.46, I²=0%); therefore, a fixed-effect model was applied. The pooled results showed that psychological interventions were associated with significantly lower STAI scores compared with routine care (MD = −1.74, 95% CI −2.96 to −0.52; P = 0.005; **Figure 5**).

**Figure 5:**
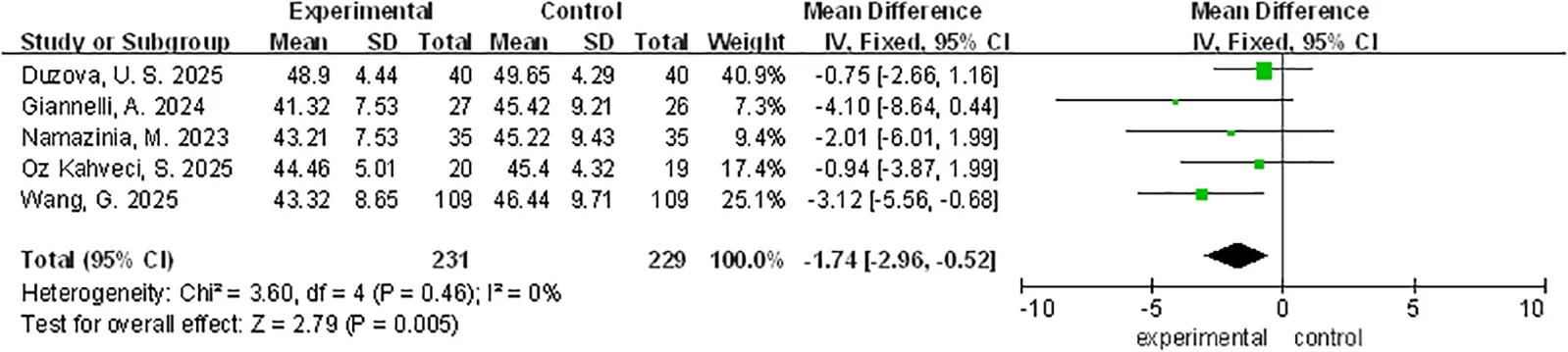
Meta-analysis of STAI comparison between psychological intervention group and routine intervention group.

### 3.5 Subgroup analysis

#### 3.5.1 Cancer type

Subgroup analysis was performed according to cancer type (**Figure 6**). In patients with breast cancer, psychological interventions were associated with significantly lower VAS scores compared with routine care (MD = −0.71, 95% CI −0.84 to −0.58; P<0.00001). A significant reduction in VAS scores was also observed in studies involving non-breast cancer patients (MD = −2.08, 95% CI −2.23 to −1.92; P<0.00001). Heterogeneity was partially reduced in the breast cancer subgroup (P = 0.03, I²=60%), whereas substantial heterogeneity persisted in the non-breast cancer subgroup (P<0.00001, I²=97%).

**Figure 6:**
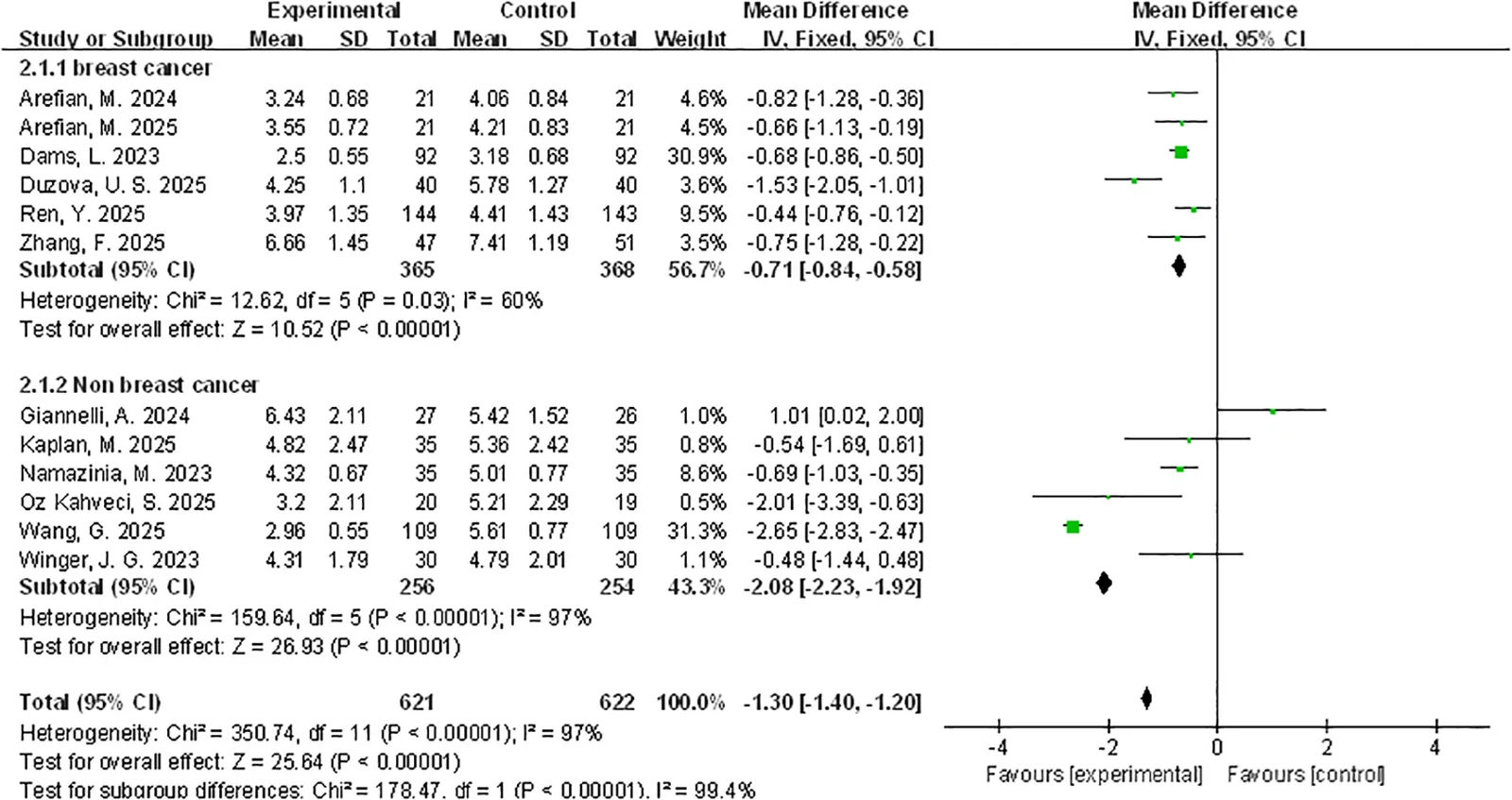
Comparison of the effects of different cancer types on VAS in patients.

#### 3.5.2 Age of patients

Subgroup analysis was also conducted according to mean patient age (**Figure 7**). In studies with a mean age <50 years, psychological interventions were associated with significantly lower VAS scores compared with routine care (MD = −0.87, 95% CI −1.22 to −0.52; P<0.00001). A significant reduction was also observed in studies with a mean age ≥50 years (MD = −0.87, 95% CI −1.57 to −0.16; P = 0.02). Heterogeneity was partially reduced in studies with a mean age <50 years (P = 0.004, I²=72%), whereas it remained substantial in studies with a mean age ≥50 years (P<0.00001, I²=98%).

**Figure 7:**
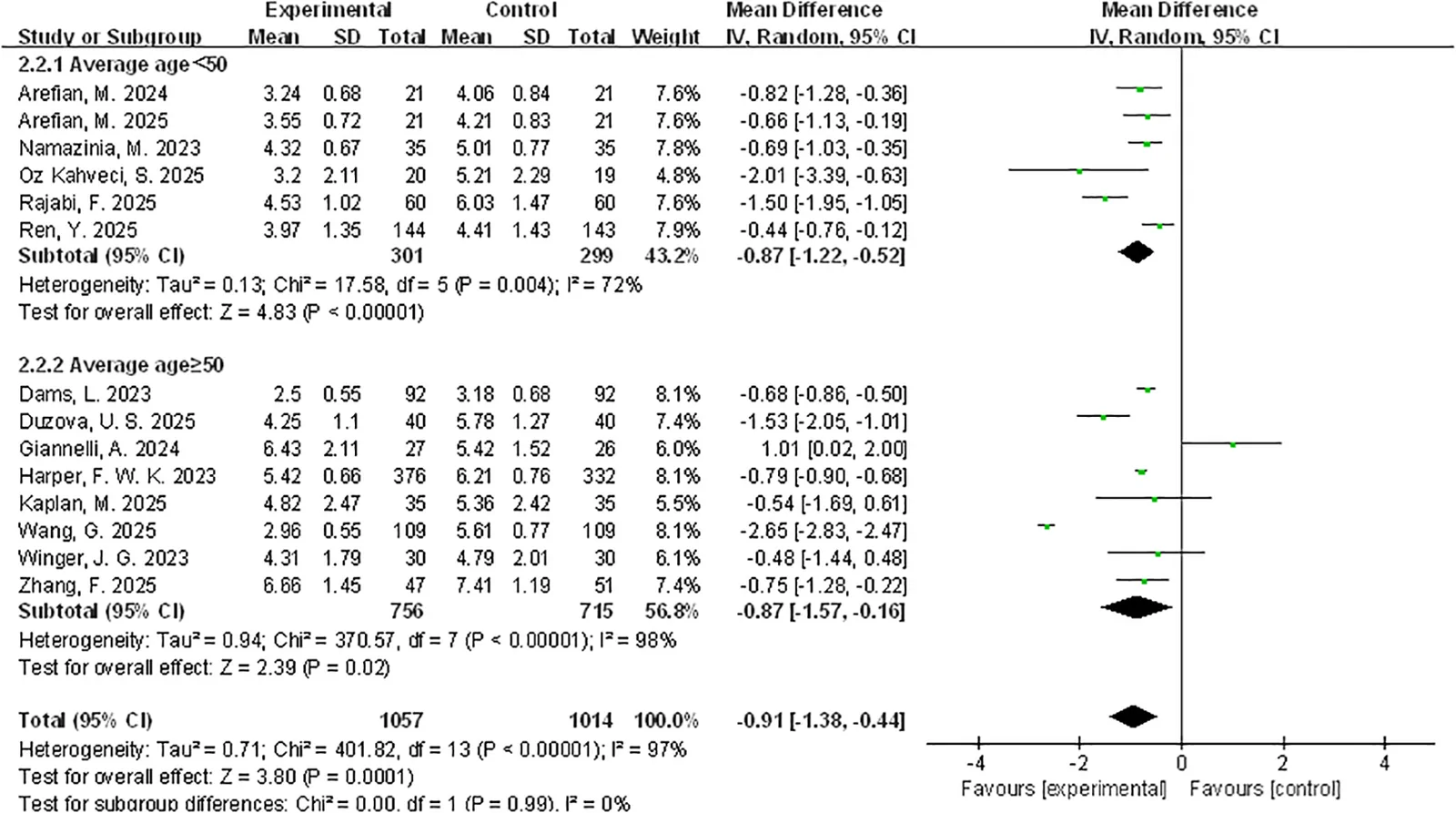
Comparison of the effects of different age on VAS in patients.

### 3.6 Publication bias

Publication bias was assessed for outcomes including at least 10 studies. For the primary outcome of VAS, the funnel plot showed that the study-specific effect estimates were generally distributed around the pooled effect, without marked asymmetry on visual assessment (**Figure 8**). Egger’s regression test further showed no statistically significant evidence of funnel plot asymmetry for VAS (intercept = 0.54, t = 0.18, P = 0.857), suggesting no significant small-study effects for the primary outcome. Because BDI included 10 studies, Egger’s regression test was also performed and showed no statistically significant funnel plot asymmetry (intercept = −0.71, t = −0.79, P = 0.452). Formal assessment of funnel plot asymmetry was not performed for STAI because only 5 studies were included. Given the limited number of studies, especially for BDI and STAI, the assessment of publication bias should be interpreted cautiously.

**Figure 8:**
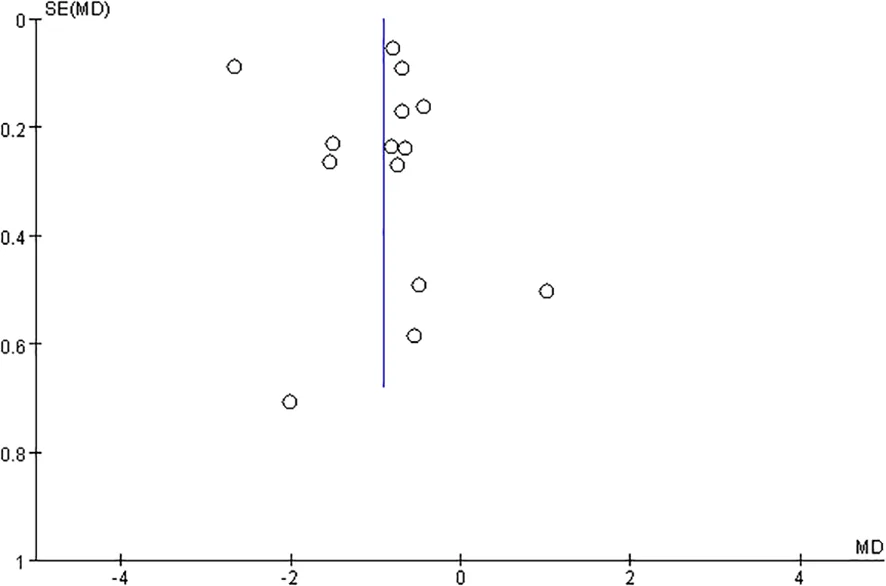
Funnel plot assessing publication bias for the VAS outcome.

### 3.7 Certainty of evidence

The GRADE assessment is summarized in **Supplementary Table 2**. The certainty of evidence was rated as moderate for all three outcomes. For VAS, 14 RCTs involving 2, 070 participants showed that psychological interventions were associated with lower pain intensity scores compared with routine care. For BDI, 10 RCTs involving 1, 664 participants showed lower depressive symptom scores in the psychological intervention group. For STAI, 5 RCTs involving 459 participants showed lower anxiety scores after psychological intervention. Overall, the current evidence suggests that psychological interventions may improve pain intensity, depressive symptoms, and anxiety in adult patients with cancer-related pain, although the findings should still be interpreted with consideration of heterogeneity and methodological limitations.

## 4 Discussion

This systematic review and meta-analysis provides an updated synthesis of randomized evidence on psychological interventions for cancer-related pain, with simultaneous evaluation of pain intensity and psychological distress. The main novelty of this study lies in integrating three clinically relevant patient-reported outcomes—VAS, BDI, and STAI—rather than focusing on pain relief alone. The pooled results showed that psychological interventions were associated with significant reductions in pain intensity, depressive symptoms, and anxiety compared with standard care, with moderate certainty of evidence across all outcomes. Clinically, these findings support the role of structured psychological interventions as adjunctive components of multimodal cancer pain management, particularly for patients in whom pain is accompanied by emotional distress (44, 45). However, the substantial heterogeneity observed for VAS indicates that intervention effects may vary according to cancer type, patient characteristics, intervention modality, duration, and contextual factors (45, 46). Therefore, psychological interventions should not be viewed as a uniform strategy but rather as patient-centered supportive approaches that require standardized protocols, appropriate patient selection, and integration with routine analgesic and oncological care (22).

The observed benefits of psychological interventions may be explained by their effects on cognitive, emotional, and neurobiological pathways involved in cancer-related pain (47). Psychological therapies may attenuate the reciprocal relationship between pain and negative affect by modifying maladaptive cognitions, reducing catastrophizing, strengthening coping strategies, and improving emotional regulation (48, 49). These mechanisms are particularly relevant because anxiety, anticipatory fear, and hypervigilance can amplify pain perception and increase symptom vigilance (50). Although the improvement in anxiety was based on fewer studies and should therefore be interpreted cautiously, it supports the clinical relevance of targeting affective components of cancer pain. Beyond psychological processes, cognitive-behavioral and mindfulness-based approaches may modulate activity in brain regions involved in pain appraisal and affective processing, including the prefrontal cortex, anterior cingulate cortex, and insula (51, 52). Such changes may enhance top-down inhibitory control of nociceptive signaling and reduce pain-related emotional amplification (53, 54). Psychological interventions may also mitigate hypothalamic–pituitary–adrenal axis dysregulation, sympathetic overactivation, and stress-related inflammatory responses, which are implicated in chronic pain persistence and cancer-related fatigue (55). These pathways are compatible with a biopsychoneuroimmunological framework in which psychological therapies may influence pain perception, emotional distress, and physiological stress responses.

The substantial heterogeneity observed for the primary pain outcome should be carefully considered when interpreting the clinical reliability of the pooled estimate. Although psychological interventions were associated with a statistically significant reduction in VAS scores, the pooled mean difference of −0.91 represents an approximately one-point improvement on a 0–10 pain scale. This effect may be modest at the individual level but clinically meaningful as an adjunctive benefit when combined with routine analgesic and oncological care. The subgroup analyses provided partial insight into the sources of heterogeneity (56). By cancer type, both breast cancer and non-breast cancer subgroups showed significant pain reduction, with an apparently larger effect in non-breast cancer studies; however, heterogeneity remained very high in the non-breast cancer subgroup, limiting definitive between-subgroup interpretation. By mean age, benefits were observed in both younger and older populations, although heterogeneity was partially reduced only among studies with a mean age <50 years. These findings suggest that cancer type and age may partly explain variability in treatment effects, but they do not fully account for the overall heterogeneity (57). Other unmeasured or inconsistently reported factors, including intervention modality, duration, delivery intensity, baseline pain severity, disease stage, concurrent anticancer treatment, follow-up timing, and cultural or healthcare-system context, may also have contributed to between-study variability (58). Therefore, while the findings are consistent with previous evidence supporting psychological approaches as adjunctive strategies for cancer pain and distress, the high heterogeneity indicates that the pooled result should be interpreted as an overall estimate across diverse interventions and populations rather than as evidence for a uniform effect applicable to all patients (59). In clinical practice, psychological interventions may be integrated as adjunctive components of multidisciplinary cancer pain management, particularly for patients with persistent pain accompanied by depressive or anxiety symptoms. However, implementation may be challenged by limited psychological resources, variability in therapist training, time constraints in oncology clinics, patient adherence, and cultural acceptance of psychological care. Future adequately powered randomized controlled trials should use standardized intervention protocols, clearly report baseline pain and psychological status, include longer follow-up, assess adherence and safety, and evaluate cost-effectiveness to clarify the feasibility and sustainability of routine clinical implementation.

Our findings are consistent with previous evidence suggesting that psychological and behavioral interventions may benefit patients with cancer. A previous meta-analysis of cognitive-behavioral and mindfulness-based interventions in advanced cancer reported small reductions in distress, supporting the relevance of psychological approaches for emotional symptom management (60). In relation to cancer pain, Ruano et al. (61) reported that psychological and non-pharmacological interventions were associated with reduced pain intensity, although the included interventions were heterogeneous. Our study extends this evidence by focusing on structured psychological interventions compared with standard care, incorporating more recent randomized trials, and evaluating pain intensity together with depressive and anxiety symptoms. In addition, the recent pilot trial by Winger et al. (42) showed that meaning-centered pain coping skills training was feasible and associated with favorable pain-related outcomes in metastatic cancer, which supports the practical relevance of integrating psychological pain-management strategies into cancer care. Nevertheless, consistent with prior literature, the substantial heterogeneity observed in our analysis indicates that intervention effects may vary across populations, intervention formats, and clinical settings.

Several limitations should be considered when interpreting these findings. First, substantial heterogeneity was observed for the primary pain outcome, which may reflect differences in intervention modality, duration, intensity, delivery format, cancer type, baseline pain severity, follow-up timing, and healthcare context across studies. Although subgroup analyses by cancer type and mean age were conducted, these factors only partly explained the heterogeneity. Second, several potentially important patient-level variables, including baseline psychological distress, analgesic use, disease stage, treatment status, and psychosocial resilience, were inconsistently reported and could not be further examined. Third, follow-up durations and outcome assessment time points varied across trials, limiting evaluation of the durability of intervention effects. Fourth, the main outcomes were based on self-reported scales, which may be influenced by expectancy effects, reporting bias, or cultural differences in symptom perception. Finally, adverse events and adherence data were not consistently reported, limiting assessment of safety and feasibility. Future studies should include adequately powered randomized controlled trials with standardized intervention protocols, clearly defined populations, consistent follow-up intervals, detailed reporting of baseline clinical and psychosocial characteristics, adherence and safety outcomes, and cost-effectiveness analyses. Individual participant data meta-analyses may further help identify patient subgroups most likely to benefit from psychological interventions.

## 5 Conclusion

This meta-analysis suggests that psychological interventions may reduce cancer-related pain intensity, depressive symptoms, and anxiety compared with standard care. However, substantial heterogeneity in the primary pain outcome limits the clinical reliability and generalizability of the pooled estimate. Therefore, these findings support psychological interventions as adjunctive components of multimodal cancer pain management rather than as a uniform strategy for all patients. Further high-quality randomized trials with standardized protocols, well-defined populations, longer follow-up, and economic evaluations are needed to strengthen the evidence for clinical implementation.

## Data Availability

The raw data supporting the conclusions of this article will be made available by the authors, without undue reservation.
