# Supplementary material for: Efficacy of psychological interventions versus standard care for cancer pain: a systematic review and meta-analysis
Source: Front Psychiatry. 2026 Sep 16;17:1811605. doi: 10.3389/fpsyt.2026.1811605 (PMC13624148; doi:10.3389/fpsyt.2026.1811605)
Supplement: Supplementary file 1 [file Table1.docx]

Supplementary Table S1.Search Strategy

| Database | Search strategy |
| --- | --- |
| PubMed | (((("Cancer Pain"[Mesh]) OR ("Neoplasm-Associated Pain"[Mesh])) OR ("cancer pain"[Title/Abstract] OR "cancer-related pain"[Title/Abstract] OR "cancer-associated pain"[Title/Abstract] OR "neoplasm-related pain"[Title/Abstract] OR "tumor-related pain"[Title/Abstract] OR "oncological pain"[Title/Abstract] OR "malignancy-related pain"[Title/Abstract])) AND ((("Psychotherapy"[Mesh]) OR ("Cognitive Behavioral Therapy"[Mesh]) OR ("Mindfulness"[Mesh])) OR ("psychological intervention*"[Title/Abstract] OR "psychosocial intervention*"[Title/Abstract] OR "cognitive behavioral therap*"[Title/Abstract] OR "acceptance and commitment therapy"[Title/Abstract] OR mindfulness[Title/Abstract] OR psychotherapy[Title/Abstract] OR "relaxation therap*"[Title/Abstract] OR "coping skills training"[Title/Abstract] OR "supportive therapy"[Title/Abstract])) AND (("Randomized Controlled Trial"[Publication Type]) OR ("randomized controlled trial"[Title/Abstract]) OR ("randomised controlled trial"[Title/Abstract]) OR ("controlled clinical trial"[Title/Abstract]) OR RCT[Title/Abstract]))) |
| Embase | ('cancer pain'/exp OR 'neoplasm pain'/exp OR 'tumor pain'/exp OR 'cancer pain':ti,ab,kw OR 'cancer-related pain':ti,ab,kw OR 'tumor-related pain':ti,ab,kw OR 'neoplasm-related pain':ti,ab,kw OR 'malignancy-related pain':ti,ab,kw) AND ('psychotherapy'/exp OR 'cognitive behavioral therapy'/exp OR 'mindfulness'/exp OR 'acceptance and commitment therapy'/exp OR 'psychological intervention*':ti,ab,kw OR 'psychosocial intervention*':ti,ab,kw OR 'supportive therapy':ti,ab,kw OR mindfulness:ti,ab,kw OR psychotherapy:ti,ab,kw) AND ('randomized controlled trial'/exp OR 'controlled clinical trial'/exp OR 'randomized':ti,ab,kw OR 'randomised':ti,ab,kw OR rct:ti,ab,kw) |
| Web of Science | TS=("cancer pain" OR "cancer-related pain" OR "cancer-associated pain" OR "neoplasm-related pain" OR "tumor-related pain" OR "oncological pain" OR "malignancy-related pain") AND TS=("psychological intervention*" OR "psychosocial intervention*" OR "cognitive behavioral therapy" OR "acceptance and commitment therapy" OR mindfulness OR psychotherapy OR "supportive therapy" OR "relaxation therapy" OR "coping skills training") AND TS=("randomized controlled trial" OR "controlled clinical trial" OR RCT OR randomized OR randomised) |
| Cochrane Library | (MeSH descriptor: [Cancer Pain] explode all trees) OR ("cancer pain" OR "tumor pain" OR "neoplasm pain" OR "malignancy-related pain"):ti,ab,kw AND (MeSH descriptor: [Psychotherapy] explode all trees OR MeSH descriptor: [Mindfulness] explode all trees OR ("psychological intervention*" OR "psychosocial intervention*" OR "cognitive behavioral therapy" OR "acceptance and commitment therapy" OR mindfulness OR psychotherapy OR "supportive therapy"):ti,ab,kw) in Trials |

Supplementary Table S2. GRADE Summary of Evidence

| Outcome | Studies / participants | Effect estimate | Heterogeneity / model | Certainty of evidence |
| --- | --- | --- | --- | --- |
| Pain intensity, VAS | 14 RCTs / 2,070 participants | MD = −0.91, 95% CI −1.38 to −0.44 | I² = 97%; random-effects | Moderate |
| Depressive symptoms, BDI | 10 RCTs / 1,664 participants | MD = −4.22, 95% CI −5.50 to −2.93 | I² = 78%; random-effects | Moderate |
| Anxiety symptoms, STAI | 5 RCTs / 459 participants | MD = −1.74, 95% CI −2.96 to −0.52 | I² = 0%; fixed-effect | Moderate |

Notes: VAS, Visual Analog Scale; BDI, Beck Depression Inventory; STAI, State-Trait Anxiety Inventory; MD, mean difference; CI, confidence interval; RCT, randomized controlled trial.
